# Glutamate dehydrogenase is a novel prognostic marker and predicts metastases in colorectal cancer patients

**DOI:** 10.1186/s12967-015-0500-6

**Published:** 2015-05-07

**Authors:** Gaojie Liu, Jie Zhu, Menglei Yu, Canfeng Cai, Yu Zhou, Min Yu, Zhiqiang Fu, Yuanfeng Gong, Bin Yang, Yingru Li, Quanbo Zhou, Qin Lin, Huilin Ye, Liangtao Ye, Xiaohui Zhao, Zhihua Li, Rufu Chen, Fanghai Han, Chaoming Tang, Bing Zeng

**Affiliations:** Department of Gastrointestinal Surgery, Sun Yat-sen Memorial Hospital of Sun Yat-sen University, Guangzhou, 510120 China; Key Laboratory of Malignant Tumor Gene Regulation and Target Therapy of Guangdong Higher Education Institutes, Sun Yat-sen Memorial Hospital of Sun Yat-sen University, Guangzhou, 510120 China; Department of Emergency Surgery, Sun Yat-sen Memorial Hospital of Sun Yat-sen University, Guangzhou, 510120 China; Department of Gastrointestinal Surgery, Qingyuan People’s Hospital, The sixth affiliated hospital of Guangzhou Medical University, Guangdong, 511518 China; Department of Pancreaticobiliary Surgery, Sun Yat-sen Memorial Hospital of Sun Yat-sen University, Guangzhou, 510120 China; Department of Pancreaticobiliary Surgery, Guangdong Academy of Medical Sciences and Guangdong General Hospital, Guangzhou, 510120 China; Department of Hepatobiliary Surgery, Cancer Center of Guangzhou Medical University, Guangzhou, 510120 China; Department of Oncology, Sun Yat-sen Memorial Hospital, Sun Yat-sen University, Guangzhou, 510120 China

**Keywords:** Colorectal cancer, Prognosis, GDH, STAT3, EMT

## Abstract

**Background:**

Glutamate dehydrogenase (GDH) is a key enzyme that catalyzes the final reaction of the glutamine metabolic pathway, and has been reported implicated in tumor growth and metastasis. However, it’s clinical significance and role in colorectal cancer (CRC) pathogenesis is largely unknown.

**Methods:**

The expression of GDH was determined by qPCR, western blot and immunohistochemistry in CRC cells and samples. The correlation of GDH expression with clinicopathologic features and prognosis was analyzed. The functional role of GDH in CRC cell proliferation, motility and metastasis was evaluated.

**Results:**

We found that GDH was up-regulated both in colorectal cancer and metastatic lesions (n = 104). Patients with high GDH expression had poorer overall survival (HR 2.32; 95% CI 1.26-4.26; *P* = 0.007) and poorer disease-free survival rates (HR 2.48; 95% CI 1.25-4.92; *P* = 0.009) than those with low GDH expression. Furthermore, we showed that GDH expression was an independent prognostic factor for CRC. In addition, over-expression of GDH promoted cell proliferation, migration and invasion in vitro, whereas loss function of GDH did the opposite. Finally, we demonstrated that the promotion of CRC progression by GDH correlated with activation of STAT3 mediated epithelial-mesenchymal transition (EMT) induction.

**Conclusions:**

These results indicate that GDH plays a critical role in CRC progression, and may provide a novel metabolism therapeutic target for CRC treatment.

**Electronic supplementary material:**

The online version of this article (doi:10.1186/s12967-015-0500-6) contains supplementary material, which is available to authorized users.

## Introduction

Colorectal cancer (CRC) is one of the leading causes of cancer mortality in most courtiers, and globally affects over a million people each year in the developed countries [1]. Distant metastasis through lymphatic or hematogenous dissemination contributes to a poor prognosis for CRC, and the liver is the most frequent site of distant metastasis of CRC [2].

Currently, liver resection remains a standard procedure and the only potentially curative therapy for colorectal liver metastases (CLM). Unfortunately, the initial resection rates are reported to be less than 25% [3], with a high recurrence rate of 70–80% after curative resection [4]. Chemotherapy alone or in addition to local minimally invasive treatment, such as radiofrequency ablation, transarterial chemotherapy, or percutaneous ethanol injection, is the most treatment options in those who are not suitable for resection [5]. However, the underlying mechanism of this aggressive biology of CRC is largely unknown.

Aberrant energy metabolism is a critical hallmark for many types of human tumors [6]. Increasingly evidences have shown that glutamine metabolism plays key roles in tumor growth and invasion, and contributes poor outcomes [7-9]. Glutamine is first catabolyzed to glutamate and than to generate a-ketoglutarate, a tricarboxylic acid (TCA) cycle intermediate. Glutaminolysis supports the viability of cancer cells by supporting ATP production and by the biosynthesis of proteins, lipids, and nucleotides, and suppress oxidative stress through glutathione synthesis [10]. More importantly, oncogenes and tumor suppressors, such as SIRT4, mTORC1, K-RAS and p53, have been implicated in the regulation of glutamine metabolism [11-14]. Accordingly, it positively regulates the mTORC1 pathway by facilitating the uptake of leucine [15], and regulates STAT3 pathway by promoting tyrosine Y705 phosphorylation [9].

Glutamate dehydrogenase (GDH) is an enzyme that plays a pivotal role in glutamine metabolism by converting glutamate to a-ketoglutarate, especially when glucose is insufficient or under hypoxia. Recently, Csibi and coworkers [16] reported that mTORC1 promoted glutaminolysis by activating GDH to facilitate cell proliferation, transformation, and tumor development by repressing SIRT4. Lorin and colleagues [15] found that GDH contributed to autophagy by activating mTORC1 and by limiting the formation of reactive oxygen species in transformed cells. Yang [17] demonstrated that GDH activity is required for glioblastoma cell to survive impairments of glycolysis brought about by glucose deprivation. Moreover, studies found that treatment with epigallocatechin gallate (EGCG), an allosteric inhibitor of GDH, has considerable effect on tumor growth [18,19]. However, the clinical significance and role of GDH expression in colorectal cancer has not yet been investigated.

In the present study, we examined the expression of GDH in CRC and further analyzed the clinical significance of GDH in a cohort of CRC patients. In addition, we explored the potential role of GDH in CRC cell proliferation and motility, which could help to better understand the pathogenesis of CRC and may further provide a novel therapeutic target for the treatment of CRC patients.

### Materials

#### Cell culture, reagents and Lentiviral transduction

The human colon cancer cell lines HCT116, DLD-1, SW480, RKO and LoVo were grown in RPMI-1640 medium supplemented with 10% fetal bovine serum (FBS). The human colon epithelial cell line NCM460 was cultured in MEM medium supplemented with 10% FBS. All cell lines were maintained at 37°C in a humidified atmosphere with 5% CO2.

AG490 and DMSO were obtained from Sigma. The GDH short hairpin RNA (shRNA) was synthesized and cloned into a pLKO.1-TRC vector (Addgene). These vectors were co-transfected into 293 T cells along with the retroviral packaging plasmid. After transfection, the supernatants were harvested and used to infect CRC cells, and the stably transfected cells were selected with puromycin according the manufacturer’s protocol.

### Tumor specimens

Twenty freshly frozen CRC samples and corresponding non tumor tissues were obtained from Sun Yat-sen Memorial Hospital of Sun Yat-sen University. In addition, we collected 104 paraffin embedded CRC specimens from our hospital between January 2002 and February 2005. Tumor staging for the specimens was carried out according to the American Joint Committee on Cancer staging criteria. The median follow-up time was 62.5 months (range from 6.7 to 99). The patients’ overall survival (OS) and disease-free survival (DFS) durations were defined as the interval from initial surgery to death and from initial surgery to clinically proven recurrence or metastasis respectively. The study was approved by the Institute Research Ethics Committee at the Sun Yat-sen University, and written informed consent was obtained from each patient.

### Immunohistochemical analysis

Sections of paraffin-embedded CRC specimens were prepared and standard immunohistochemical procedures were carried out as previously described [20]. Briefly, samples were deparaffinized and rehydrated, and the endogenous peroxidase activity was quenched. Antigen retrieval was performed, and the sections were blocked with bovine serum albumin and then incubated with anti-GDH antibody (Abcam; 1:200). Sections were washed and then incubated with a biotinylated secondary antibody and visualized with 3,3-diaminobenzidine.The staining results were scored by two pathologists blinded to the clinical data. Staining index was calculated as the product of the staining intensity (0, no staining; 1, weak staining; 2, moderate staining; 3, strong staining) and the proportion of positive cells (0, no positive tumor cells; 1, <10%; 2, 10-35%; 3, 35-70%; 4, >70%). The immunoreactivity score (IRS) was resulted from the multiplication of both parameters. Using this method of assessment, we evaluated GDH expression by determining the IRS, with scores of 0, 1, 2, 3, 4, 6, 8, 9 or 12. The samples were divided into 2 groups as follows: low (IRS = 0–4), and high (IRS ≥6).

### RNA extraction and quantitative real-time PCR

Total RNA was extracted using TRIzol reagent. The reverse-transcription PCR (RT-PCR) was performed using transcriptase, and the quantitative real-time PCR (qRT-PCR) was performed in a LightCycler480 System using a SYBR Premix ExTaq kit according to the manufacturer’s instructions. Primers for qRT-PCR are as follows. GDH: Forward, 5′-GGG ATT CTA ACT ACC ACT TGC TCA-3′, Reverse 5′-AAC TCT GCC GTG GGT ACA AT-3′. GAPDH: Forward, 5′-CTC CTC CTG TTC GAC AGT CAG C-3′, Reverse, 5′-CCC AAT ACG ACC AAA TCC GTT-3′. The relative expression levels were calculated by the 2^-ΔΔ^CT method. Each assay was carried out in triplicate.

### Western blot analysis

Cell cytosolic protein fractions were prepared using RIPA buffer. According to standard Western blot procedures, briefly, proteins were separated by SDS-PAGE and then transferred to polyvinylidene fluoride (PVDF) membranes. After blocking in 5% nonfat milk, the membranes were incubated with the following primary antibodies: GDH antibody (Abcam), STAT3, pSTAT3 (Tyr705), E-Cadherin, Vimentin, ZEB1 and GAPDH antibody (Cell Signaling Technology) according to the manufacturer’s instructions.

### Cell proliferation

Cell proliferation was analysed with the MTT assay. Cells were seeded in 96-well plates at a density of 1,000 cells per well. At 1, 2, 3 and 4 days, the cells were stained with 20 μl of MTT (0.5 mg/ml) for 4 h, and after which the medium was removed, and 100 μl of DMSO was added. The absorbance was measured at 490 nm. The anchorage-independent sphere formation assay was performed by culturing the cells in suspension in serum-free DMEM-F12 supplemented with B27 (Invitrogen), EGF (BD Biosciences, CA, USA), 0.4% bovine serum albumin (Sigma, MO, USA), and insulin (Sigma).

### Cell migration and invasion assays

Cell motility was assessed by wound healing assay as previously described [21]. Results were expressed as a migration index: the distance migrated by targeted relative to the distance migrated by control cells. Cell invasion assays were performed using 24-well transwells (8-μm pore size, BD Sciences) coated with matrigel (1 mg/ml, BD Sciences), as previously described [21]. The inserts were stained with 0.2% crystal violet, imaged, and counted under an inverted microscope in six randomly selected fields. All experiments were carried out in triplicate and repeated at least three times.

### Statistical analysis

Data are presented as the mean ± SD, and differences between groups were analyzed using Student’s t-test or chi-squared test or fisher exact test. The Kaplan-Meier method and log-rank test were used to estimate survival rates. Cox proportional hazards model was used to calculate univariate and multivariate hazard ratios for the study variables. Statistical analyses were performed with SPSS 16.0 software (Chicago, IL), and P values of < 0.05 were considered statistically significant.

## Results

### Expression of GDH in CRC cells and tissues

**Figure 1:**
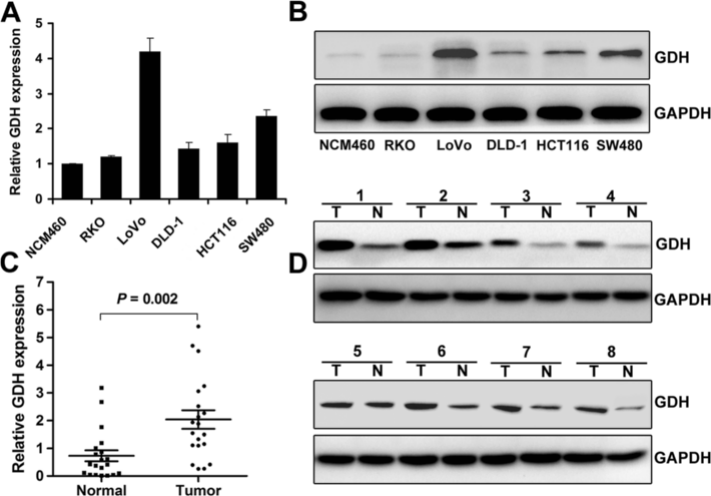
Expression levels of GDH in CRC cell lines and clinical samples. **(A-B)** Expression levels of GDH mRNA and protein in NCM460 and CRC cell lines. **(C-D)** Expression levels of GDH mRNA and protein in CRC tissues and corresponding non tumor tissues. GAPDH was used as the endogenous control. Data are presented as the mean ± SD, and *P* values were calculated with Student’s t-test.

Quantitative RT-PCR and western blot were used to determine the levels of GDH mRNA and protein in CRC cell lines. As shown in Figure 1A and B, GDH was over-expressed in CRC cell lines when compared to human colon epithelial cell line NCM460. LoVo (*P* = 0.0003), HCT116 (*P* = 0.0036), HCT116 (*P* = 0.0019). Next, we analyzed GDH mRNA expression in 20 freshly frozen CRC tissues and corresponding non tumor tissues. We found that GDH expression levels were considerably over-expressed in CRC when compared to corresponding non tumor tissues (Figure 1C). Moreover, GDH protein expression levels were also up-regulated in tumor tissues (Figure 1D).

### GDH over-expression is associated with CRC metastasis

**Figure 2:**
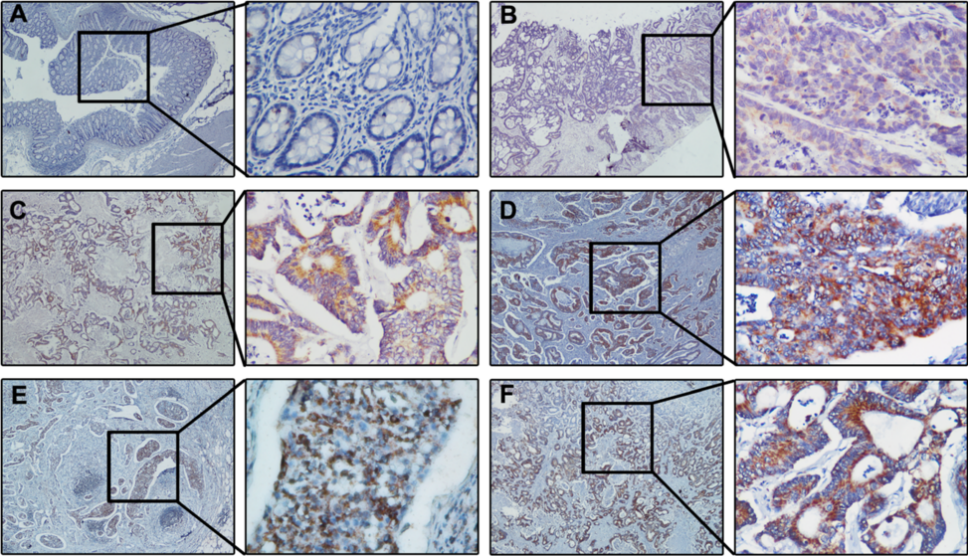
Expression levels of GDH in CRC tissues, metastatic lymph nodes and liver metastases lesions. Representative images of CRC tissues with GDH Negative staining **(A)**, Weak staining: light yellow **(B)**, moderate staining: yellow brown **(C)**, strong staining: brown **(D)**. Representative images of GDH immunostaining in metastatic lymph nodes **(E)** and liver metastases lesions **(F)**. (Envision × 40, × 400).

To determine the effect of GDH expression on CRC progression, we next analyzed the expression of GDH protein in a set of 104 paraffin embedded CRC tissue using immunohistochemistry. Representative staining of GDH in CRC tissue is shown in Figure 2. We observed that GDH was highly expressed in 56 of the 104 (53.8 %) patients, and GDH-positive staining was mainly in the cytoplasm of the cancer cells. Interestingly, immunostaining results showed that metastatic lymph nodes and liver metastases lesions had higher levels of GDH expression (Figure 2E and F).

Furthermore, patients with high GDH expression exhibited a significant association with tumor size (*P* = 0.046), tumor stage (*P* = 0.001), lymph node metastasis (*P* = 0.001) and liver metastasis (*P* = 0.021). While there were no significant associations between GDH expression and age, sex, location, grade of differentiation, T stage, CEA, venous invasion, or nervous invasion (Table 1).

**Table 1:** **Clinicopathological factors and GDH expression in 104 colorectal cancers**

**Factor**	**GDH high expression**		**GDH low expression**		***P*** **-value**
	**n = 56, n (%)**		**n = 48, n (%)**		
Age					
<60	27	48.2	26	54.2	0.545
≥60	29	51.8	22	45.8	
Gender					
Male	34	60.7	27	56.2	0.645
Female	22	39.3	21	43.8	
Location					
Colon	33	58.9	32	66.7	0.416
Rectum	23	41.1	16	33.3	
Tumor size					
<3cm	24	42.9	30	62.5	0.046*
≥3cm	32	57.1	18	37.5	
CEA					
Normal	26	46.4	25	52.1	0.656
Elevated	30	53.6	23	47.9	
Grade of differentiation					
Well	11	19.6	14	29.2	0.296
Moderate	35	62.5	27	56.3	
Poor	10	17.9	7	14.5	
Depth of invasion					
T1 and T2	31	55.4	26	54.2	0.903
T3 and T4	25	44.6	22	45.8	
Tumor stage					
I and II	14	25	28	58.3	0.001*
III and IV	42	75	20	41.7	
Lymph node metastasis					
Negative	20	35.7	33	68.8	0.001*
Positive	36	64.3	15	31.2	
Liver metastasis					
Negative	40	71.4	43	89.6	0.021*
Positive	16	28.6	5	10.4	
Venous invasion					
Negative	50	89.3	45	93.8	0.501
Positive	6	10.7	3	6.2	
Nervous invasion					
Negative	36	64.3	38	79.2	0.095
Positive	20	35.7	10	20.8	

### GDH over-expression is association with poor prognosis in CRC patients

**Figure 3:**
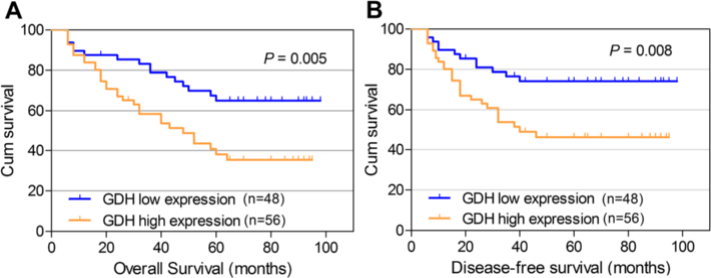
Kaplan-Meier curves for OS and DFS. Patients with high GDH expression had poorer overall survival **(A)** and poorer disease-free survival **(B)** rates than patients with low GDH expression.

To assess the clinical significance of GDH over-expression in CRC, Kaplan-Meier analysis and the log-rank test were used to analyze the relationship between GDH expression and patient survival. We found that the 5-year overall survival (OS) was significantly lower in patients with high GDH expression than in those with low GDH expression (38.1% vs. 65.1%; HR 2.32; 95% CI 1.26-4.26; *P* = 0.007) (Figure 3A). Moreover, patients with high GDH expression were significantly associated with poorer disease-free survival (DFS) rates than those with low GDH expression (HR 2.48; 95% CI 1.25-4.92; *P* = 0.009) (Figure 3B). In addition, multivariate analyses indicated that GDH expression, lymph node metastasis, and liver metastasis were independent prognostic indicators for both OS and DFS in CRC patients (Table 2).

**Table 2:** **Univariate and multivariable Cox regression analyses for overall and disease-free survival**

**Factor**	**Univariate analysis**				**Multivariate analysis**			
	**HR**	**95% CI**		***P*** **-value**	**HR**	**95% CI**		***P*** **-value**
**Overall survival**								
Depth of invasion (T1-2/T3-4)	2.07	1.16	3.69	0.014*	1.82	0.98	3.38	0.060
Tumor stage (I-II/III-IV)	2.01	1.11	3.63	0.021*	1.37	0.72	2.6	0.333
Grade of differentiation (well, moderate/poor)	2.13	1.01	4.45	0.046*	1.28	0.53	3.05	0.581
Lymph node metastasis (negative/positive)	4.68	2.45	8.94	<0.001*	3.47	1.66	7.27	0.001*
Liver metastasis (negative/positive)	3.53	1.88	6.63	<0.001*	2.62	1.29	5.32	0.008*
Venous invasion (negative/positive)	2.45	1.04	5.8	0.042*	1.66	0.64	4.32	0.294
Nervous invasion (negative/positive)	2.25	1.25	4.05	0.007*	1.14	0.59	2.19	0.703
GDH (low/high)	2.32	1.26	4.26	0.007*	2.42	1.23	4.78	0.011*
**Disease-free survival**								
Lymph node metastasis (negative/positive)	5.73	2.68	12.28	<0.001*	4.70	2.08	10.59	<0.001*
Liver metastasis (negative/positive)	3.81	1.95	7.46	<0.001*	2.38	1.16	4.87	0.018*
GDH (low/high)	2.48	1.25	4.92	0.009*	2.17	1.05	4.47	0.037*

*HR* relative risk, *95% CI* 95% confidence interval.

*Statistically significant *P*<0.05, Cox proportional hazard regression model.

### GDH knockdown impairs CRC cell proliferation and motility in vitro

**Figure 4:**
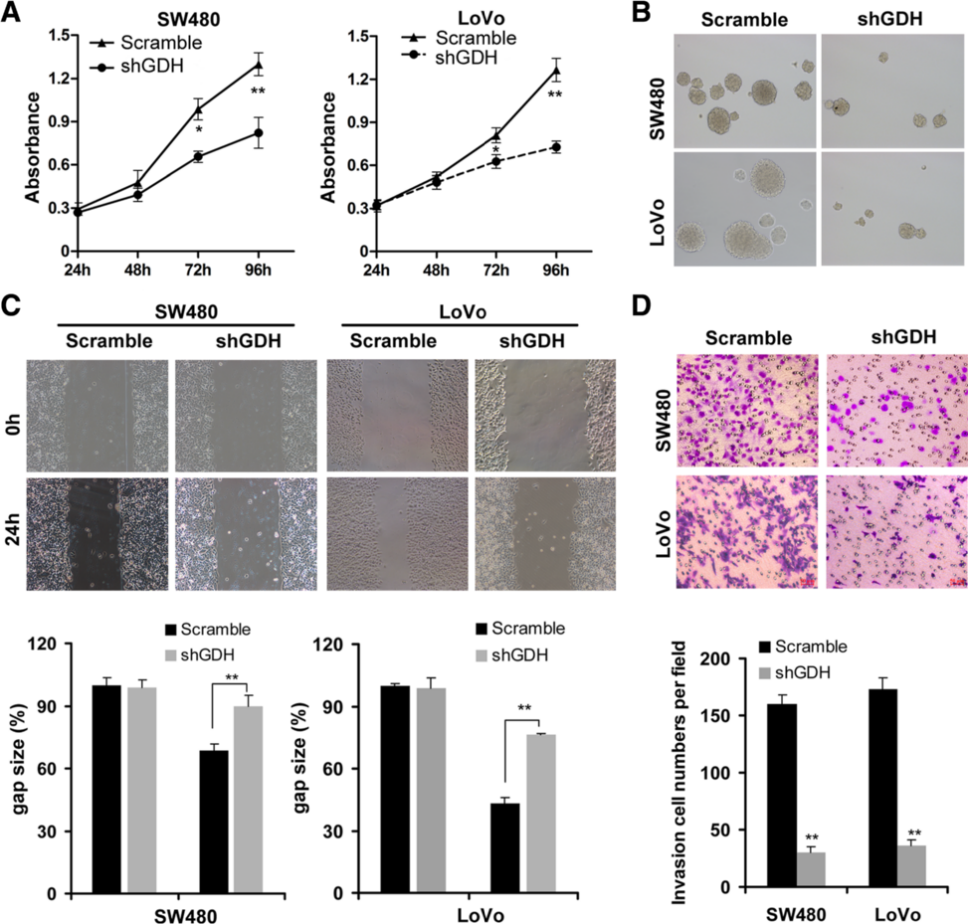
GDH knockdown impairs CRC cell proliferation and motility. Effects of GDH knockdown on cell proliferation **(A)**, anchorage-independent growth **(B)**, migration **(C)** and invasion **(D)** of SW480 and LoVo cells. Data are presented as the mean ± SD based on three independent experiments. *P < 0.05, **P < 0.01 compared to the control using Student’s t-test.

To confirm the functional role of GDH in CRC cells, we first transfected shGDH or scrambled plasmid into CRC cells (Additional file 1: Figure S1), and found that GDH depletion significantly suppressed cell proliferation and inhibited anchorage-independent growth compared to the controls (Figure 4A and B). Next, the wound healing assay showed that down-regulation of GDH led to a marked decrease in cell motility than that of control cells (Figure 4C). Similarly, the transwell invasion assay demonstrated that GDH depletion resulted in a significant reduction of cell invasion (Figure 4D).

### GDH promotes CRC cell motility via STAT3 mediated EMT Induction

**Figure 5:**
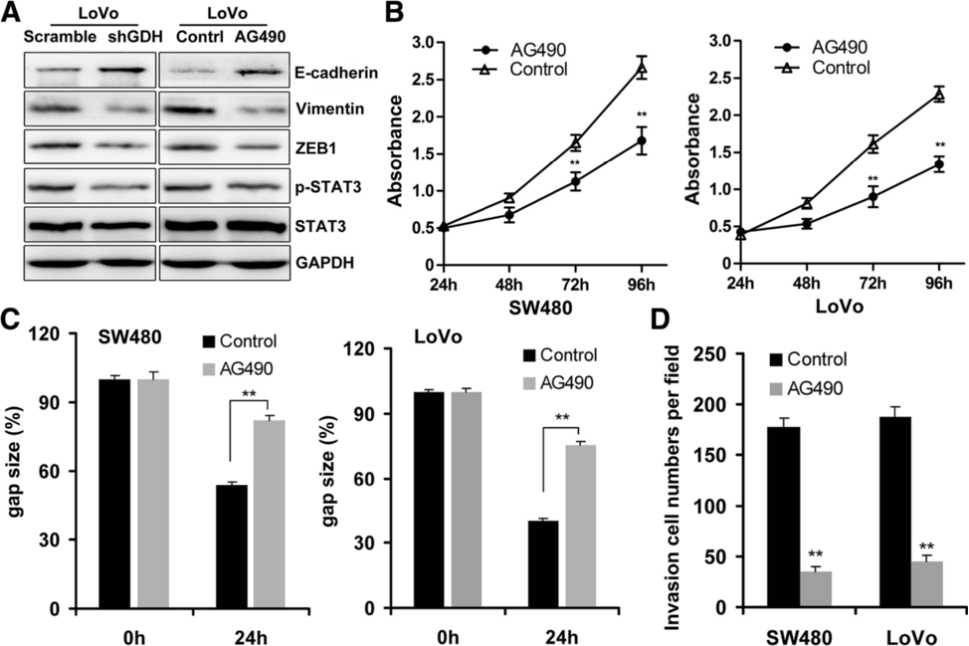
GDH promotes CRC cell motility via STAT3 mediated EMT Induction. **(A)** Effects of GDH knockdown and AG490 on STAT3 Tyr705 phosphorylation, ZEB1 Vimentin and E-cadherin protein expression in CRC cells detected by western blot analysis. **(B-D)** Effects of GDH knockdown on cell proliferation, migration and invasion of CRC cells. Data are presented as the mean ± SD based on three independent experiments. *P < 0.05, **P < 0.01 compared to the control using Student’s t-test.

Epithelial-mesenchymal transition (EMT) contributes to tumor invasion and metastasis in many cancers including CRC [22]. Previous studies found that glutamine metabolism could promote tumor growth and invasion through STAT3 pathway, a regulator of EMT and aberrantly activated in CRC [9]. These studies prompted us to ask whether GDH promotes CRC cell motility through STAT3 mediated EMT Induction. Interesting, we found that knockdown of GDH significant attenuated STAT3 phosphorylation, and decreased Vimentin and ZEB1 expression, while up-regulated E-cadherin expression in CRC cells (Figure 5A). Furthermore, blocked STAT3 pathway by AG490 significantly increased E-cadherin and decreased ZEB1 and Vimentin expressions in CRC cells (Figure 5A). Additionally, STAT3 pathway blockage could significant inhibited cell proliferation (Figure 5B), and attenuated the migration and invasion ability of CRC cells (Figure 5C and D).

To further prove this hypothesis, we establish GDH-overexpression SW480 cells, and found that GDH-overexpression could promote STAT3 phosphorylation, and up-regulate Vimentin and ZEB1 expression, while decrease E-cadherin expression (Additional file 2: Figure S2). Furthermore, AG490 treatment could significantly increase E-cadherin, and decrease ZEB1 and Vimentin expressions in GDH-overexpression SW480 cells (Additional file 2: Figure S2). Taken together, these results suggested that GDH promotes CRC cell motility via STAT3 mediated EMT induction.

## Discussion

Distant metastasis is a major cause of death in CRC patients [23]. In this study, we found that GDH was up-regulated in both CRC cells and clinical samples, and correlated with poor survival and liver metastasis of CRC. Importantly, our results revealed that GDH is an independent prognostic factor for survival, which might served as valuable prognosis markers for CRC. Finally, we demonstrated that GDH promotes CRC cell motility via STAT3 mediated EMT Induction. We show here for the first time that GDH plays important roles in the metastatic and aggressive biology of CRC, which might served as a predictive marker for CRC prognosis, and presents a viable metabolic regulation strategy for CRC.

Metabolism is now recognized as a key feature of the cancer cells, and increasingly evidence has linked cell metabolism with cancer outcome [24-26]. Although intensive studies have focused on the role of glutamine metabolism in regulating growth and survival [27-29], its role in motility and metastasis of cancers is not well understood. Recently, Yang [9] revealed that high invasive OVCA cells were strongly glutamine dependent, and glutamine was a key mitochondrial substrate for driving cancer metastasis by induction of STAT3 serine phosphorylation. Moreover, glutamine restriction inhibited melanoma cell migration via modulation of actin cytoskeleton remodeling [30], and GDH activity was required for glioblastoma cell to survive impairments of glycolysis brought about by glucose deprivation [17]. In the present study, we focus on the role of GDH, a key enzyme converting glutamate to a-ketoglutarate then entry into the TCA cycle. We found a high expression of GDH in aggressive CRC cell lines, and altered GDH expression affected cell proliferation, migration and invasion. More importantly, we found direct clinical w?>evidence of a strong correlation between GDH over-expression and unfavorable clinicopathologic variables such as tumor size, tumor stage, lymph node metastasis and liver metastasis. These findings robustly suggested that GDH plays an important role in CRC progression and metastasis.

It has been reported that approximately 30% of CRC patients presenting with synchronous metastases and 70 % ultimately developing liver metastases, and 50% of CRC deaths are caused by liver metastases [31-33]. However, the mechanism of this aggressive biology of CRC is largely unknown. Epithelial-mesenchymal transition (EMT) involves the loss of E-Cadherin mediated cell adhesion and polarity, and is proposed to be a crucial mechanism regulating the initial steps in metastatic progression [34]. Several studies have examined the possible role of EMT in CRC progression including liver metastases [35-37]. To our knowledge, few studies have described EMT with glutamine metabolism in CRC. In this study, we found that loss function of GDH lead to aberrant expression of EMT markers, and suppressed cell proliferation and motility of CRC cells. Together with previous studies, our findings further support the crucial role of EMT in CRC metastasis.

Evidences showed that metabolism enzyme themselves could be oncogenic by altering cell signaling that mediated multiple aspects of cancer metastatic biology [15,28,38]. Considering the crucial role of GDH involved in CRC metastases, it is necessary to elucidate the underlying mechanism of GDH mediated induction of EMT. STAT3, a key regulator of EMT, has been reported frequently activated in CRC [39-41]. Zhou [42] showed that STAT3 could induce PTTG expression to facilitate tumor growth and metastasis of CRC. Xiong [43] found that STAT3 down-regulated E-Cadherin expression via ZEB1 in colorectal cancer cells. In this study, we demonstrated that altered GDH expression significantly affected the tyrosine phosphorylation of STAT3, and GDH mediated invasiveness of CRC was STAT3 depended. Our results consist with previous findings that tyrosine phosphorylation levels of STAT3 are dependent on glutamine’s entry into the TCA cycle.

## Conclusions

In conclusion, this study provided evidence supporting the critical role of GDH in CRC progression and metastasis. Our findings uncover a novel molecular mechanism for CRC progression and metastasis and provide novel metabolism therapeutic targets and strategies to control CRC progression and metastasis.

## Additional files

Additional file 1:
**Effects of GDH knockdown in CRC cells.**
Additional file 2:
**GDH promotes EMT via STAT3 pathway.**

## Abbreviations

CRC: Colorectal cancer
GDH: Glutamate dehydrogenase
EMT: Epithelial-mesenchymal transition
CLM: Colorectal liver metastases
OS: Overall survival
DFS: Disease-free survival
TCA cycle: Tricarboxylic acid cycle
