# Supplementary figures and images for: Glutamate dehydrogenase is a novel prognostic marker and predicts metastases in colorectal cancer patients
Source: J Transl Med. 2015 May 7;13:144. doi: 10.1186/s12967-015-0500-6 (PMC4490642; doi:10.1186/s12967-015-0500-6)

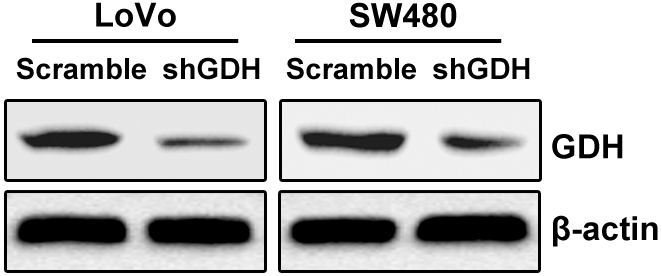

Supplement: Additional file 1: — Effects of GDH knockdown in CRC cells. [file 12967_2015_500_MOESM1_ESM.tiff]

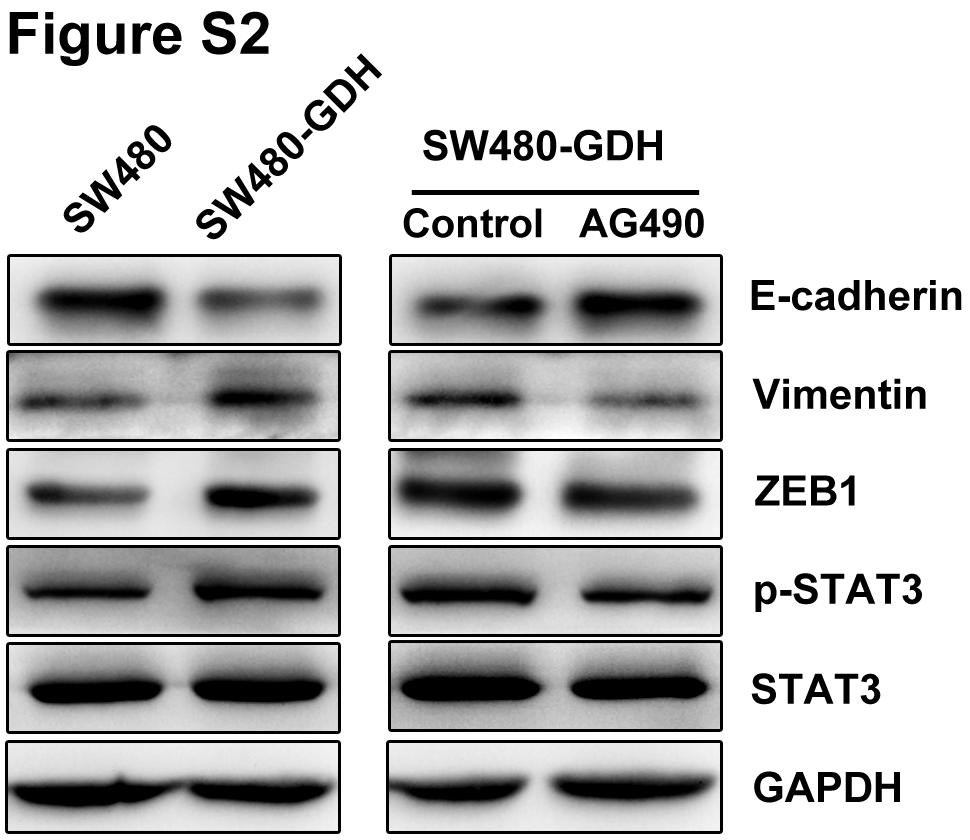

Supplement: Additional file 2: — GDH promotes EMT via STAT3 pathway. [file 12967_2015_500_MOESM2_ESM.tiff]
